# Oxytocin Receptor Polymorphisms are Differentially Associated with Social Abilities across Neurodevelopmental Disorders

**DOI:** 10.1038/s41598-017-10821-0

**Published:** 2017-09-14

**Authors:** Danielle A. Baribeau, Annie Dupuis, Tara A. Paton, Stephen W. Scherer, Russell J. Schachar, Paul D. Arnold, Peter Szatmari, Rob Nicolson, Stelios Georgiades, Jennifer Crosbie, Jessica Brian, Alana Iaboni, Jason Lerch, Evdokia Anagnostou

**Affiliations:** 1 Department of Psychiatry, University of Toronto, Toronto, Ontario, Canada; 2 Department of Biostatistics Design and Analysis, The Hospital for Sick Children, Dalla Lana School of Public Health, University of Toronto, Toronto, Ontario, Canada; 3 The Centre for Applied Genomics, The Hospital for Sick Children, Toronto, Ontario, Canada; 4 The McLaughlin Centre, University of Toronto, Toronto, Ontario, Canada; 5 Department of Psychiatry, The Hospital for Sick Children, Toronto, Ontario, Canada; 6 Hotchkiss Brain Institute, Departments of Psychiatry & Medical Genetics, University of Calgary, Calgary, Alberta Canada; 7 The Centre for Addiction and Mental Health, Toronto, Ontario, Canada; 8 The Children’s Health Research Institute and Western University, London, Ontario, Canada; 9 Department of Psychiatry and Behavioural Neurosciences, McMaster University, Chedoke Hospital, Hamilton, Ontario, Canada; 10 Department of Paediatrics, University of Toronto, Toronto, Ontario Canada; 11 Autism Research Centre, Bloorview Research Institute, Holland Bloorview Kids Rehabilitation Hospital, Toronto, Ontario, Canada; 12 Program in Neuroscience and Mental Health, The Hospital for Sick Children, Department of Medical Biophysics, University of Toronto, Toronto, Canada

## Abstract

Oxytocin is a pituitary neuropeptide that affects social behaviour. Single nucleotide polymorphisms (SNPs) in the oxytocin receptor gene (*OXTR*) have been shown to explain some variability in social abilities in control populations. Whether these variants similarly contribute to the severity of social deficits experienced by children with neurodevelopmental disorders is unclear. Social abilities were assessed in a group of children with autism spectrum disorder (ASD, n = 341) or attention deficit hyperactivity disorder (ADHD, n = 276) using two established social measures. Scores were compared by *OXTR* genotype (rs53576, rs237887, rs13316193, rs2254298). Unexpectedly, the two most frequently studied *OXTR* SNPs in the general population (rs53576 and rs2254298) were associated with an increased severity of social deficits in ASD (p < 0.0001 and p = 0.0005), yet fewer social deficits in ADHD (p = 0.007 and p < 0.0001). We conclude that these genetic modifier alleles are not inherently risk-conferring with respect to their impact on social abilities; molecular investigations are greatly needed.

## Introduction

The past decade has yielded major advances in our understanding of the genetics of human social behaviour. Twin and family studies have shown that traits such as empathy and social cognition have moderate to high heritability^1, 2^. Genome wide association studies (GWAS) and candidate gene studies now seek to identify specific underlying genes and polymorphisms that influence social abilities^1^. Oxytocin and vasopressin are pituitary neuropeptides that exert robust effects on social processes in animal models^3, 4^. In humans, it is hypothesized that these peptides may influence social behaviour by enhancing the saliency of social cues, or by attenuating fear/stress^5^. Plasma oxytocin levels (whether endogenous, or exogenously administered) have been shown to correlate with facial emotion recognition abilities^6–8^. In control populations, common polymorphisms in oxytocin or vasopressin receptor genes (*OXTR* and *AVPR1a)* have been associated with differences in empathy^9–11^, prosocial temperament^12^, social sensitivity^13–17^, and stress reactivity in social contexts^18, 19^. The area on chromosome 3 carrying the *OXTR* gene (3p24–26) has emerged on GWAS with respect to risk for autism spectrum disorder (ASD) at a genome wide significance level^20^. Meta-analyses indicate that certain neuropeptide variants are more common in individuals with ASD than in controls^21^, and may influence social abilities in the general population^22^.

ASD and attention deficit hyperactivity disorder (ADHD) are common childhood onset neurodevelopmental disorders that are highly heterogeneous, present along a spectrum of severity, and often comorbid^23, 24^. Both disorders are associated with social deficits, inattention, and hyperactivity to varying degrees^23–25^, leading some to propose that they may be different manifestations of an overarching disorder^23^. In addition to rare genetic mutations and environmental factors, the additive effects of common genetic variants are hypothesized to contribute to the development of these disorders^26^, and may explain some of the observed heterogeneity in behaviour, abilities, and psychopathology. Preliminary investigations suggest that single nucleotide polymorphisms (SNPs) in *OXTR* may act as modifier alleles with respect to the severity of social deficits experienced by individuals with ASD (For a review of the literature, see Supplementary Table S1). Two studies have also examined *OXTR* SNPs in small groups of children with ADHD^27, 28^. It remains unclear whether *OXTR* SNPs act as: 1) general risk factors for social impairment across populations (i.e. effects span diagnostic boundaries), or 2) specific modifier alleles in certain behavioural or biological context (i.e. disorder specific effects). Two recent studies have shown that *OXTR* variants were associated with a similar magnitude and direction of social differences in children with ASD and their unaffected relatives^6, 29^. This question, however, has not yet been examined across different diagnostic groups, where the environmental, behavioural, and genetic architecture differ. Given the increasing number of studies employing trans-diagnostic approaches in psychiatry^30^, it is important to examine whether behavioural risk variants function similarly across disorders.

In this study, we examined the association between four SNPs in *OXTR* and social abilities in a large sample of children and youth with ASD or ADHD and present the first cross disorder comparison of genotype effects. To extend on preliminary research to date, we used two validated continuous social metrics, and restricted initial hypotheses to Caucasian participants only, given evidence of possible ancestral differences in the effects of *OXTR* on social abilities^31, 32^. First, in our group of children with ASD, we confirmed several associations between social deficits and *OXTR* variants (*OXTR* rs53576 G-allele, rs237887 A-allele, and rs2254298 A-allele) that had been previously shown in smaller samples. New associations were detected in the group of children with ADHD. Second, we compared the magnitude and direction of the impact of common variation in *OXTR* on social abilities between diagnostic groups (ASD vs. ADHD). Based on the family studies described above, and the high degree of symptom overlap and comorbidity between ASD and ADHD, we hypothesized that genotype effects would not differ by diagnosis. However, unexpectedly, highly significant diagnostic differences were detected, with effectively a reciprocal direction of association between genotype and social abilities between the two diagnostic groups. Our findings indicate that common polymorphisms in neuropeptide receptor genes likely represent a specific and not a general risk modifier, with effects that vary by diagnosis in the context of different biological, behavioural, or environmental conditions. These results have implications for all analyses involving common alleles and complex traits.

## Results

### Study sample

The sample consisted of 617 participants with either ASD or ADHD (n = 404 Caucasian). The ASD group had lower mean intelligence quotients (IQs), and higher anxiety levels (see Table 1). Genotype frequencies and linkage analyses are presented in Supplementary Tables S2 and S3, compared to/based on reference genome data from the 1000 Genomes Project^33^. *OXTR* allele frequencies did not deviate from Hardy Weinberg equilibrium (Supplementary Table S4), except for rs237887 in ADHD.

**Table 1:** Demographic data.

	n (%)	Mean Age in Years (SD)	Females n (%)	Mean IQ (SD)	Mean CBCL Anxiety T-score (SD)	Mean SCQ Score (SD)	Mean SCQ SocCom (SD)	Mean RMET Incorrect Items (SD)
**All Participants**								
**ASD**	341	10.6						
*Caucasian*	211 (62.8)	11.3 (3.8)	48 (22.8)	87.7 (25.9)	64.1 (8.7)			
*Non-caucasian*	130 (38.1)	9.8 (4.0)	25 (19.2)	77.1 (27.0)	64.1 (9.2)			
**ADHD**	276							
*Caucasian*	193 (69.9)	10.7 (2.8)	46 (23.8)	100.9 (16.7)	60.6 (8.7)			
*Non-caucasian*	83 (30.1)	10.0 (2.3)	15 (18.1)	98.8 (13.8)	60.3 (8.4)			
ASD vs. ADHD*		p = 0.2	p = 0.9	p < 0.0001	p < 0.0001			
**Participants with SCQ Scores**								
**ASD**	332							
*Caucasian*	207	11.3 (3.8)	48 (23.2)	87.5 (26.0)		19.2 (7.5)	13.6 (6.3)	
*Non-caucasian*	125	9.9 (4.0)	24 (19.2)	76.9 (27.1)		20.1 (7.7)	14.7 (6.5)	
**ADHD**	274							
*Caucasian*	192	10.7 (2.9)	46 (24.0)	100.9 (16.7)		6.7 (5.1)	5.0 (3.9)	
*Non-caucasian*	82	10.0 (2.4)	15 (18.3)	98.5 (13.9)		8.2 (5.5)	5.7 (4.0)	
ASD vs. ADHD*		p = 0.1	p = 0.9	p < 0.0001		p < 0.0001	p < 0.0001	
**Participants with RMET Scores, Age** > **6, and IQ Scores**								
**ASD**	190							
*Caucasian*	131	12.1 (3.3)	29 (22.1)	96.6 (20.0)				11.4 (4.5)
*Non-caucasian*	59	11.8 (3.2)	10 (17.0)	88.6 (21.2)				14.0 (5.2)
**ADHD**	109							
*Caucasian*	73	9.5 (1.9)	15 (20.6)	101.5 (16.2)				11.6 (4.0)
*Non-caucasian*	36	9.6 (2.2)	6 (16.7)	98.4 (14.5)				12.1 (3.4)
ASD vs. ADHD*		p < 0.0001	p = 0.8	p = 0.004				p = 0.4

IQ: Intelligence Quotient, CBCL: Child Behavior Checklist, SCQ: Social Communication Questionnaire, Soc Com: Social communication and interaction items only (repetitive behaviour items excluded), RMET: Reading the Mind in the Eyes Test, ASD: Autism spectrum disorder, ADHD: Attention deficit hyperactivity disorder. Analyses using the RMET were adjusted for IQ, therefore only participants with an RMET score and IQ data were included for these analyses. *P-values from ANOVA’s comparing demographic variable between ASD and ADHD, adjusted for ancestry.

### Measures

Severity of social deficits were quantified by examining the number of incorrect items selected by the participants on the Reading the Mind in the Eyes Test (RMET)^34^, and through their parent/caregiver’s ratings of concerns on the Social Communication Questionnaire (SCQ) (see methods)^35^. Therefore, throughout the manuscript, higher scores on either metric suggest greater social deficits. Correlations between measures in ASD and ADHD are shown in Supplementary Tables S5 and S6. Twelve percent of the participants with ADHD had SCQ scores that fell above the cut-off for concern for ASD (Total SCQ > 15). For comparison, 23% of the participants with ASD fell above the cut-off for clinical concern regarding ADHD on the Child Behavior Checklist (T-score > 70).

### Aim 1

#### Confirmation of previous *OXTR* genotype effects on social abilities

Using logistic regression, we initially examined *OXTR* SNPs with prior evidence of genotype effects on social abilities within each diagnostic group (in ASD: rs53576, rs2254298, and rs237887; in ADHD: rs53576 and rs13316193), restricting the sample to those of Caucasian ancestry only, and adjusting for other possible confounding factors (see Methods). When considering multiple testing, five comparisons (3 in ASD and 2 in ADHD) across two social metrics yielded a corrected p-value of 0.005 as the threshold for significance for an overall alpha of 0.05.

In the Caucasian ASD cohort, we hypothesized that the *OXTR* rs53576 G-allele carriers, rs2254298 A-allele carriers, and the rs237887 AA carriers would have greater social deficits, based on previous literature. In line with our hypotheses, rs53576 G-allele carriers (GG/GA) had higher SCQ scores [Odds Ratio (OR) of an increase in SCQ score for the high-risk genotype group compared to low-risk genotype group: 1.4 (95% CI: 1.2–1.6), Z-value = 4.2, p < 0.0001 uncorrected]. Differences were non-significant on the RMET for this SNP (Z = 1.2, p = 0.2) (Table 2, left panel). Similarly, for *OXTR* rs2254298, the hypothesized high-risk group (AA/AG) also had higher SCQ scores in the ASD cohort (OR 1.2, 95% CI: 1.1–1.4, Z = 3.5, p = 0.0005), suggesting greater social deficits (Table 2). There were no differences on the RMET (Z = −1.2, p = 0.2). As hypothesized, *OXTR* rs237887 genotype was associated with differences in social abilities, with the AA-allele group having greater social deficits than the GG group on the both the SCQ (OR: 1.2, 95% CI: 1.0–1.4) and the RMET (OR: 1.2, 95% CI: 1.0–1.5), although neither finding would survive correction for multiple comparisons (SCQ: p = 0.05; RMET: p = 0.06; Table 2).

**Table 2:** Aim 1: Association between *OXTR* SNP genotype and social deficits on the SCQ and RMET scores

	Caucasian Only					All Ancestry				
	n High-/ Low-risk Group	Hypothesized High-risk Group Mean (95% CI)	Hypothesized Low-risk Group Mean (95% CI)	p value	OR High- vs. Low-risk	n High-/ Low-risk Group	Hypothesized High-risk Group Mean (95% CI)	Hypothesized Low-risk Group Mean (95% CI)	p value	OR High- vs. Low-risk
	**ASD**					**ASD**				
**rs53576**		**GG/GA**	**AA**				**GG/GA**	**AA**		
SCQ	180/27	13.8 (13.4–14.3)	11.5 (10.5–12.5)	<0.0001^*^	1.4 (1.2–1.6)	293/39	14.6 (14.2–15.0)	12.5 (11.6–13.4)	<0.0001	1.4 (1.2–1.5)
RMET	114/17	11.2 (10.7–11.8)	10.4 (9.2–11.7)	0.2	1.1 (0.9–1.4)	168/22	11.9 (11.3–12.4)	10.8 (9.7–12.0)	0.09	1.2 (1.0–1.4)
**rs2254298**		**AA/GA**	**GG**				**AA/GA**	**GG**		
SCQ	48/159	14.7 (14.0–15.5)	13.2 (12.8–13.7)	0.0005^*^	1.2 (1.1–1.4)	82/250	15.3 (14.7–15.9)	14.1 (13.7–14.5)	0.0005	1.2 (1.1–1.3)
RMET	26/105	10.6 (9.5–11.6)	11.3 (10.7–11.8)	0.2	0.9 (0.8–1.1)	36/154	10.9 (9.9–11.8)	11.9 (11.4–12.5)	0.03	0.9 (0.7–1.0)
**rs237887**		**AA**	**GG**				**AA**	**GG**		
SCQ	71/40	14.5 (13.8–15.2)	13.4 (12.6–14.3)	0.05	1.2 (1.0–1.4)	109/58	14.8 (14.2–15.4)	13.5 (12.8–14.3)	0.004	1.2 (1.1–1.4)
RMET	48/21	11.6 (10.7–12.5)	10.3 (9.1–11.5)	0.06	1.2 (1.0–1.5)	70/27	12.2 (11.4–13.0)	10.6 (9.5–11.8)	0.01	1.3 (1.1–1.5)
**rs13316193**		**CC**	**TT**				**CC**	**TT**		
SCQ	22/82	13.3 (12.1–14.5)	12.7 (12.1–13.3)	0.4	1.1 (0.9–1.3)	37/129	14.5 (13.6–15.4)	13.9 (13.3–14.4)	0.2	1.1 (0.9–1.3)
RMET	17/46	11.5 (10.1–12.8)	10.9 (10.1–11.7)	0.4	1.1 (0.9–1.4)	23/70	12.5 (11.2–13.7)	11.8 (11.1–12.5)	0.3	1.1 (0.9–1.4)
	**ADHD**					**ADHD**				
**rs53576**		**GG/GA**	**AA**				**GG/GA**	**AA**		
SCQ^†^	171/21	4.5 (4.1–4.8)	5.6 (4.8–6.6)	0.007	0.7 (0.6–0.9)		N/A^†^	N/A^†^		
RMET	67/6	10.9 (10.2–11.7)	13.5 (11.3–15.7)	0.02	0.7 (0.5–1.0)	100/9	11.0 (10.4–11.7)	13.4 (11.6–15.2)	0.01	0.7 (0.5–0.9)
**rs2254298**		**AA/GA**	**GG**				**AA/GA**	**GG**		
SCQ	51/141	3.5 (3.0–4.0)	5.0 (4.6–5.3)	<0.0001	0.7 (0.6–0.8)		N/A^†^	N/A^†^		
RMET	21/52	10.8 (9.7–12.0)	11.2 (10.4–12.0)	0.5	0.9 (0.8–1.1)	32/77	10.9 (9.9–11.9)	11.3 (10.6–12.1)	0.4	0.9 (0.8–1.1)
**rs237887**		**AA**	**GG**				**AA**	**GG**		
SCQ	73/44	4.8 (4.3–5.3)	5.4 (4.8–6.1)	0.08	0.9 (0.7–1.0)	90/54	5.0 (4.5–5.6)	5.5 (4.8–6.2)	0.2	0.9 (0.8–1.1)
RMET	27/17	11.5 (10.4–12.7)	12.0 (10.5–13.4)	0.6	0.9 (0.7–1.2)	36/19	12.1 (10.8–13.3)	12.5 (10.8–14.4)	0.6	0.9 (0.7–1.2)
**rs13316193**		**CC**	**TT**				**CC**	**TT**		
SCQ^†^	19/83	5.3 (4.5–6.4)	4.7 (4.3–5.2)	0.2	1.2 (0.9–1.5)		N/A^†^	N/A^†^		
RMET	8/36	12.2 (10.2–14.2)	11.7 (10.6–12.7)	0.6	1.1 (0.8–1.4)	9/51	12.3 (10.3–14.3)	11.7 (10.7–12.7)	0.6	1.1 (0.8–1.4)

SCQ: Social Communication Questionnaire, score out of 28 social communication/ interaction items only, RMET: Reading the Mind in the Eyes Test, number of incorrect items out of 28, ASD: Autism spectrum disorder, ADHD: Attention deficit hyperactivity disorder. All analyses were adjusted for age, sex, and those involving the RMET were adjusted for IQ. LS mean: least squares mean adjusted to age 11.0. The high-risk/ low-risk groupings were based on the patterns observed in previous studies. OR: Odds Ratio of an increased score on an item on the SCQ/RMET for the high-risk genotype group compared to low-risk genotype. P-values are uncorrected. Asterisks indicate survival after correction for multiple comparisons^†^.Cross indicates ancestry by genotype interaction was significant (p < 0.05), therefore data were not pooled across ancestry groups.

In the Caucasian ADHD cohort, we hypothesized that *OXTR* rs13316193 CC genotype group would have greater social deficits, as would the rs53576 G-allele carriers. In our sample, *OXTR* rs13316193 genotype was not associated with differences in social abilities on either metric (SCQ: Z = 1.2, p = 0.2; RMET: Z = 0.5, p = 0.6, Table 2). *OXTR* rs53576 genotype was associated with differences on both the SCQ (Z = −2.7, p = 0.007) and RMET (Z = −2.3; p = 0.02); the direction of deficits was congruent between metrics [OR for SCQ: 0.7 (95% CI: 0.6–0.9); OR for RMET: 0.7 (95% CI: 0.5–1.0)], yet opposite to the direction hypothesized, and detected in the ASD group.

**Figure 1:**
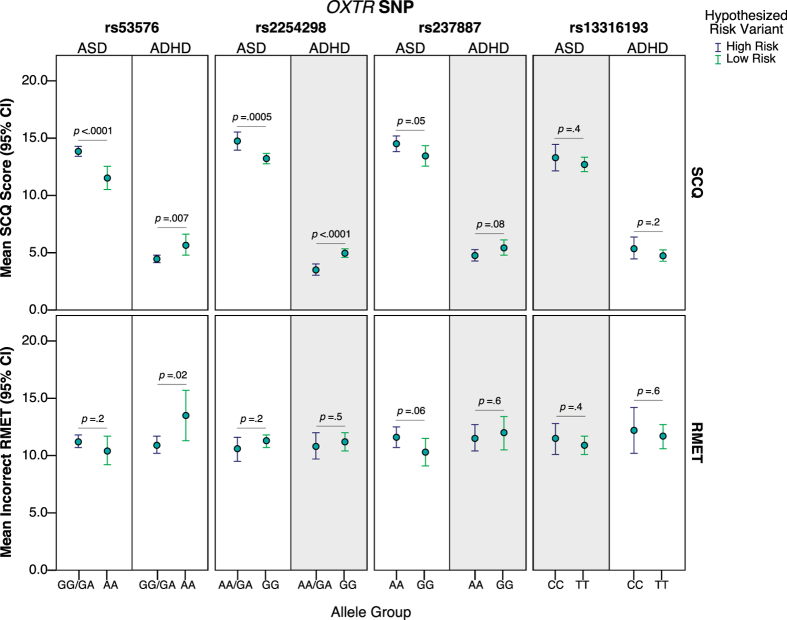
Comparison of the association between *OXTR* genotype and social deficits between diagnostic groups in participants with Caucasian ancestry. Predicted scores at age 11. SCQ: Social Communication Questionnaire- score out of 28 social communication/interaction items only, RMET: Reading the Mind in the Eyes Test, number of incorrect items out of 28, ASD: Autism spectrum disorder, ADHD: Attention deficit hyperactivity disorder. Higher scores across both metrics suggest greater social deficits. All analyses were adjusted for age and sex differences, and those involving the RMET were adjusted for IQ as well. The high-risk/ low-risk groupings were based on the patterns observed in previous studies. P-values are uncorrected. Error bars represent 95% confidence intervals (CI). White panels indicate primary hypotheses from the literature (aim 1). Exact sample sizes detailed in Tables 1 and 2.

The results from our primary hypotheses are displayed in Fig. 1 (white panels); values are indicated in Table 2 for genotype risk group comparisons, and mean scores across all genotypes are shown in Supplementary Table S7. Covariates from these categorical logistic regression models in the Caucasian cohort are displayed in Supplementary Tables S8 and S9. In summary, several previous associations between *OXTR* SNPs and social abilities were confirmed in the group of children with ASD, but not ADHD.

### Aim 2

#### Examination across diagnostic groups

Next, we combined data across both diagnostic groups, and tested for genotype*diagnosis interactions in the Caucasian participants. After correcting for multiple comparisons (n = 8), a p-value of 0.006 was used for significance regarding the interaction term. In this context, we also tested for genotypic differences in social abilities across all SNPs in the remaining diagnostic groups (i.e. those not previously tested as part of our primary aims, including *OXTR* rs2254298 and rs237887 in ADHD, as well rs13316193 in ASD) (Fig. 1, grey panels, Table 2).

For rs53576, the diagnostic interaction terms were significant on both the SCQ and the RMET (SCQ: Wald χ^2^ = 25.0, p < 0.0001; RMET: Wald χ^2^ = 11.4, p = 0.0007), indicating that the ‘risk variant’ in ASD showed a significantly different effect in ADHD (as shown above, the OR of social deficits with the hypothesized risk variant was >1 on both metrics in ASD, but <1 on both metrics in ADHD, see Table 2). For *OXTR* rs2254298, the genotype*diagnosis interaction term was significant for the SCQ only (Wald χ^2^ = 31.8, p < 0.0001). In ADHD, the AA/GA-allele was associated with *fewer* social deficits on the SCQ [Z = −4.7; p < 0.0001, OR 0.7 (0.6–0.8)], opposite to the direction initially detected in the ASD group (OR = 1.2 in ASD). The diagnostic interaction term for *OXTR* rs2254298 was not significant on the RMET (Wald χ^2^ = 0.3, p = 0.6), where the risk allele had no significant effect in ADHD or ASD (Table 2). Likewise, there were no significant differences in social abilities on either metric for rs13316193 in ASD nor in ADHD (Table 2), resulting in non-significant SNP*diagnosis interactions. Although no significant allele effects were found for rs237887 after correcting for multiple comparisons (Table 2), the trend towards effects in opposite directions across the two diagnoses resulted in similarly borderline interaction effects across both outcomes (SCQ: Wald χ^2^ = 5.9, p = 0.02; RMET: Wald χ^2^ = 3.0, p = 0.08). In summary, across multiple *OXTR* variants, there was evidence that the hypothesized risk allele was associated with more significant social deficits in ASD, but fewer social deficits in ADHD.

### Secondary aims

As secondary aims, we also examined (1) whether social abilities varied by arginine vasopressin receptor 1a *(AVPR1a)* RS3 microsatellite length, and (2) the impact of ancestry on *OXTR* findings. Greater length of the *AVPR1a* RS3 microsatellite was previously associated with greater receptor expression levels and superior social abilities in typically developing populations^36–38^, although no effect was detected in a large sample of children with ASD^29^. Previous studies have also found evidence for a differential direction of effects of *OXTR* polymorphisms in Caucasian vs. non-Caucasian individuals, particularly for rs2254298^31, 32^.

### (1) *AVPR1a* RS3

*AVPR1a* is the gene coding for the corresponding vasopressin receptor. Upstream of the exon is a microsatellite promoter region, which includes an RS3 segment of CT, TT, and GT repeats. The RS3 segment varies in length; in our sample the range was 310–346 base pairs (bp), with a median of 328. We categorized each individual’s promoter length as ‘long’ or ‘short, with the cut-point placed at 328 bp, as this was both the median in our sample, and similar to cut-points used in previous studies (e.g. 326 and 328^36^, 326^39^, or median value^40^,). We considered those carrying two ‘short’ alleles to be the high-risk group, and compared them to those with two ‘long’ alleles as the low-risk group. In the group of Caucasian participants with ASD, there were no differences in social abilities based on microsatellite length on the SCQ [short/short vs. long/long, Z = −0.9, p = 0.3, OR = 0.9 (95% CI: 0.8–1.1)], or the RMET [Z = 1–0.6, p = 0.1, OR = 0.8 (95% CI: 0.7–1.0)]. Similarly, in the ADHD group, there were no differences in social abilities by microsatellite length on the SCQ [Z = 0.7, p = 0.4, OR = 1.1 (0.9–1.2)] or on the RMET [Z = −0.6, p = 0.6, OR: 0.9 (0.7–1.2)].

### (2) Examination of *OXTR* across ancestry groups

We subsequently combined data across Caucasian and non-Caucasian participants, and tested for ancestry*genotype interactions. The aim was to determine whether the direction of genotype effects was sufficiently similar between the Caucasian and non-Caucasian groups to justify performing the genotype group comparisons on the entire combined sample, irrespective of ancestry. For the ASD cohort, ancestry*genotype interaction terms were non-significant for all *OXTR* SNPS, on the SCQ and on the RMET (p > 0.05 for all). In the ADHD group, there were no significant ancestry*genotype interactions on the RMET (p > 0.05 for all), but significant effects were observed on the SCQ for rs2254298 (Wald χ^2^ = 29.1, p < 0.0001), where the opposite direction of an association between the risk allele and social deficits was detected in the non-Caucasian cohort [OR = 1.4 (95% CI: 1.1–1.7), p = 0.002] compared to the Caucasian cohort [OR = 0.7 (95% CI: 0.6–0.8) p < 0.001]. Interaction terms were also significant in ADHD for rs53576 (Wald χ^2^ = 7.6, p = 0.006) and rs13316193 (Wald χ^2^ = 5.1, p = 0.02) on the SCQ.

For SNPs where the ancestry*genotype interactions were conservatively non-significant (p > 0.05), we pooled data across ancestry groups, and calculated the combined odds ratios in the larger sample (Table 2, right side). Overall, the direction of effects was very similar in the combined and Caucasian only samples, although the larger combined sample size led to more significant associations.

### Exploratory analyses

The finding of differential effects of genotype on social deficits between diagnostic groups was unexpected. To further explore and confirm this pattern, we repeated the analyses using additive genotype risk models (GG vs. GA vs. AA), as opposed to categorical (GG/GA vs. AA). Results were similar with either model (see Supplementary Table S10).

Next, given an emerging hypothesis that oxytocin may affect social behaviour by decreasing anxiety or fear responses, potentially at the level of the amygdala^41, 42^, and given the high rate of comorbid anxiety disorders in ASD (up to 40%)^43^, we post-hoc hypothesized that differential anxiety levels between the two clinical groups could potentially explain the reciprocal pattern. Anxiety was quantified through parent report on the Child Behavior Checklist anxiety subscale (CBCL-anxiety) and the Revised Children’s Anxiety and Depression Scale (RCADS). We compared anxiety scores by diagnosis, then by genotype, and subsequently included anxiety as an additional covariate in the original SNP-SCQ models. Anxiety analyses were restricted to participants who also had SCQ scores available and were of Caucasian ancestry (CBCL n = 377, RCADS n = 235), to facilitate comparison to previous findings.

On the CBCL-anxiety, the ASD group had higher mean T-scores (Table 1) and higher total anxiety raw scores compared to the ADHD group [mean raw score = 4.4 (95% CI: 3.5–4.6) vs. 2.9 (2.1–3.8), t = −4.0, p < 0.0001]. On the RCADS, we found higher social anxiety in the ASD group compared to the ADHD group [mean T-score of 58.0 (54.5–61.5) vs. 53.1 (48.6–57.6), t = −2.2, p = 0.03]. There were no differences in total anxiety on the RCADS between diagnostic groups [52.9 (49.8–56.0) vs. 51.9 (47.8–55.9), p = 0.6]. Results were unchanged after adjusting for age and sex.

For genotype group comparisons, we tested for effects of rs53576 and rs2254298 on anxiety measures in ASD and ADHD separately. No genotype effects were detected on the RCADS for either SNP (p > 0.05). For rs53576, a modest genotype effect was detected on the CBCL-anxiety raw score in the ASD group in line with the direction of effects seen with the SCQ [AA = 2.9 (95% CI: 1.8–4.0); GG/GA = 4.6 (4.2–5.1), t = −2.9, p = 0.004]. There were no significant effects of rs53576 on CBCL-anxiety in the ADHD group (difference: −0.2, p = 0.7). The SNP*diagnosis interaction term was non-signification (p = 0.09), suggesting a lack of differential effects of genotype on anxiety between the two diagnostic groups. For rs2254298, CBCL-anxiety scores were modestly affected by genotype in the ADHD group only (AA/GA vs. GG: −1.0 points, p = 0.03). The diagnostic interaction term was again non-significant (p = 0.3).

We subsequently included CBCL-anxiety score as an additional covariate in the SCQ models for rs53576 and rs2254298. Although CBCL-anxiety was significantly associated with SCQ in the overall model, results regarding the effects of *OXTR* on SCQ scores were otherwise unchanged [rs53576 in ASD: OR = 1.4 (1.1–1.7), rs53576 in ADHD: OR = 0.8 (0.6–0.9), rs53576*dx: p < 0.0001; rs2254298 in ASD: OR = 1.3 (1.1–1.5), rs2254298 in ADHD: OR = 0.7 (0.6–0.8), rs2254298*dx: p < 0.001]. Overall, data suggest that *OXTR* genotype may affect anxiety to some extent, but a reciprocal pattern between diagnostic groups as seen on social measures was not observed, and anxiety did not explain the differential effects of genotype on social outcomes by diagnosis.

## Discussion

In this study, we examined how common polymorphisms in neuropeptide receptor genes affected social abilities in a large group of children and youth with ASD or ADHD. This is the first study comparing genotype effects across two neurodevelopmental disorders, with the largest ADHD cohort, and one of the largest ASD cohorts for this type of analysis to date. Other strengths of this study include: participants from two clinical groups who were recruited and characterized using the same research protocol; detailed phenotyping on both participant and caregiver completed metrics; and examination for potential ancestral differences in genotype effects. Our main findings were: 1) confirmation of several previous associations regarding neuropeptide receptor genotype and social abilities in our large group of children with ASD, 2) a lack of replication of previous associations between *OXTR* SNPs and social abilities seen in smaller ADHD cohorts, although several new associations were detected in this larger sample, and 3) significant diagnostic differences in the association between *OXTR* genotype and social abilities (i.e. a genetic modifier allele associated with more social deficits in ASD had either no effect, or conferred a benefit with respect to social abilities in ADHD).

Our findings are in line with direction of effects described in previous ASD cohorts and family studies. For example, Parker *et al*. examined genotypic differences in social abilities in a group of 79 children with ASD, 53 unaffected siblings, and 62 controls, of varied ethnicities^6^. They found that carriers of the A-allele of *OXTR* rs2254298 had higher levels of social impairment, and carriers of the G-allele of *OXTR* rs53576 performed worse on an affect recognition task^6^. Skuse *et al*. tested for associations between 60 *OXTR* SNPs and 31 *AVPR1a* SNPs and social abilities in an ASD cohort of 198 families from the United Kingdom and Finland, of mostly Caucasian ancestry^29^. Only *OXTR* rs237887 genotype affected face recognition memory, where AA carriers performed significantly worse. Length of *AVPR1a* RS3 microsatellite promoter region did not impact social abilities in their sample^29^, nor in ours. A recent meta-analysis found both the *OXTR* rs237887 A-allele and the rs2254298 A-allele were also risk-conferring with respect to an ASD diagnosis^21^. It would follow that in certain vulnerable families, specific *OXTR* common variants may push a small subset of those at risk over the diagnostic threshold.

Few studies have examined the impact of *OXTR* on social functioning in ADHD^27, 28^. Park *et al*. found that rs53576 AA allele carriers had fewer social deficits in their sample of 119 participants. However, in our ADHD sample, *OXTR* rs53576 GG allele carriers and rs2254298 A allele carriers had fewer social deficits, opposite to the direction of effects seen in our ASD group. For *OXTR* rs53576, this finding may be more in keeping with patterns observed in control populations. In a recent meta-analysis of mostly typically developing adults, rs53576 G-allele homozygotes had better social abilities on a variety of metrics^22^. Similarly, a previous study using the RMET found that healthy adolescents who had the *OXTR* rs53576 GG genotype had higher accuracy overall^18^. Our data suggest that the direction of impact of common variants in these receptors vary by disorder- a protective variant in one condition may increase risk in another. Overall, this would indicate that common polymorphisms in *OXTR* represent a specific vulnerability factor, and not a universal risk factor with respect to social functioning.

Mechanistically, it may be that these non-coding intronic SNPs co-localize with specific receptor haplotypes, other important exons, or certain rare variants^44^, that affect the structure or function of *OXTR*. Alternatively, they may be markers of functional differences in the non-coding regulatory elements of neuropeptide receptor genes, indirectly affecting receptor expression levels. SNPs in non-coding regulatory elements were recently shown to exert a robust effect on oxytocin receptor expression levels within specific brain regions in prairie voles^45^. Similar investigations in humans are lacking and are greatly needed. At the same time, large and rare genetic variants may be more common in ASD, and could be potentially associated with more significant disruptions on oxytocin signaling^46, 47^. For example, contactin-associated protein 2 (*CNTNAP2*) mutations have been associated with ASD and developmental delay^46^. A recent mouse model of ASD using *CNTNAP2* knockout showed disrupted oxytocin expression and social deficits, which were corrected with exogenous oxytocin administration^47^. While usually we conceptualize rare variants as acting on a background of common variants, the implication of our study is genetic background may also play a critical role in modifying the impact of common ‘risk’ variants on behavioural phenotypes.

How exactly similar changes in receptor expression or function could result in disparate behavioural consequences in different diagnostic groups is unknown. Possible explanations include: 1) specific neurocircuits contributing to social deficits may vary between the disorders, 2) other diagnostic differences in environmental, hormonal, or epigenetic processes may be differentially accentuated through oxytocin signaling or 3) measurement factors or confounding.

With respect to neurocircuitry, emerging literature indicates that social and fear perception involve complex networks centred on the amygdala and reward centres^42, 48^, and that oxytocin may potentially enhance transmission in these circuits through its effects on interneurons^41, 49^. It may be that the brain circuits, regions, or underlying behavioural constructs contributing to differences in SCQ scores may vary between diagnostic groups. While our exploratory analyses involving anxiety measures suggested that *OXTR* genotype may also impact on anxiety levels to some extent, a reduction in anxiety alone was insufficient to explain the differential *OXTR* effects across diagnostic groups.

Alternatively, it is possible that other diagnostic differences in environmental, hormonal, or epigenetic processes may be differentially accentuated through oxytocin signaling. For example, several studies in typically developing cohorts have shown that the *OXTR* rs53576 G-allele was associated with increased environmental sensitivity, which led to more adverse outcomes under conditions of early childhood adversity or exclusion^13–17^. G-allele carriers were also shown to experience a more beneficial response to receiving positive social support as indicated by salivary cortisol levels^50^, and were more likely to seek social support under stress^51^. In this context, perhaps children with ASD are less likely to experience the stress buffering effects of social contact given their underlying social deficits, putting the more ‘sensitive’ allele group at greater risk. At the same time, there is evidence to suggest that oxytocin may play a role in suppressing the cortisol stress response^52^. Given evidence of divergent cortisol patterns in ASD vs. ADHD (i.e. enhanced cortisol responses to stress in ASD^53^, and attenuated cortisol profiles in ADHD^54, 55^), it would follow that an *OXTR* variant leading to increased cortisol activity would be disadvantageous in ASD, but potentially advantageous in ADHD. Differential epigenetic regulation of *OXTR* gene expression between the two disorders could also potentially explain diagnostic differences in SNP effects (for a review see ref. 56). While there is evidence to suggest that increased methylation of certain *OXTR* sites may be associated with increased social deficits and autistic traits in ASD^57, 58^, and callous/ unemotional traits in ADHD^59, 60^, to our knowledge, epigenetic profiles of *OXTR* have not been compared between the two disorders.

Measurement factors and confounding could also potentially contribute to observed diagnostics differences. While testing protocols were identical across diagnostic groups, it is possible that our social measures may differentially capture certain aspects of sociality in ASD vs. ADHD (e.g. attachment security, social reward, or other more complex social tasks). While common confounding factors were adjusted for in our analyses, the role of clinical severity, adaptive functioning, and parental stress or psychopathology were not explored, and could potentially affect response patterns on either social metric. The impact of different traits and measurement factors on SCQ scores in ASD and ADHD in particular merits further exploration.

In terms of other limitations, despite our sample size, we may have been underpowered to detect more subtle differences, including interactions with ancestry or analyses on the smaller group with RMET and IQ scores. Lack of statistical significance would therefore not necessarily rule out a possible biological effect of genotype on social behaviour for these measures. Analyses including a comorbid ASD/ADHD group, a control group, or a group of unaffected family members, would have proven interesting comparisons but were not feasible with the existing dataset. While results were statistically significant, differences between genotype groups were generally small and not meant to imply clinical utility. Human molecular/ expression data is greatly needed to clarify the mechanisms through which these variants may influence receptor function and downstream behaviours.

In summary, our findings indicate that common polymorphisms in neuropeptide receptor genes act as specific (as opposed to general) modifier alleles regarding social phenotypes in children with neurodevelopmental disorders, with effects that vary by diagnosis. Our findings therefore contribute to the often-conflicting literature regarding these SNPs. We are the first to implicate potential diagnostic differences as one explanation for heterogeneity in findings. Future studies comparing *OXTR* genotype effects across these disorders ought to examine the role of the hypothalamic pituitary axis social neurocircuitry, environmental social stress, and rare genetic, or epigenetic differences as possible mechanisms. Molecular studies investigating the functional impacts of these SNPs in humans are also greatly needed.

## Methods

### Participants and study protocol

Participants were recruited via the Province of Ontario Neurodevelopmental Disorders (POND) Network, a coordinated multi-centre research initiative examining the neurobiology of neurodevelopmental disorders. Hospital Research Ethics Boards approved the study protocol at each participating institution (Holland Bloorview Kids Rehabilitation Hospital, Toronto; The Hospital for Sick Children, Toronto; McMaster Children’s Hospital, Hamilton; and Lawson Health Research Institute, London); all experiments were performed in accordance with relevant guidelines and regulations. Informed consent was obtained from parents of all participants; further details of the protocol are described elsewhere^25^. All participants (n = 614) were between 4 and 21 years of age, and had a clinical diagnosis of ASD or ADHD (see Table 1). Clinical diagnoses were confirmed via in-depth diagnostic assessments using established measures. Ancestry was determined by parent/caregiver self-report on the ethnic origins of the participants’ biological four grandparents. Where all four grandparents were identified as Caucasian (including Scandinavian, Ashkenazi Jewish, French Canadian, Mediterranean, Eastern European, Russian, Western European), the participant was classified as ‘Caucasian.’ Participants with one or more grandparents of non-Caucasian ancestry were classified as ‘non-Caucasian.’

### Quantitative behavioural phenotype

The Social Communication Questionnaire (SCQ) is a validated measure completed by a parent/caregiver to screen for ASD^35, 61^, developed based on the items and factors in the Autism Diagnostic Interview-Revised^62^. Higher SCQ scores (out of 39) indicate a greater risk, with a cut-off of > 15 proposed to distinguish between those with and without ASD^35^. For context, typically developing children usually score under 10 on the SCQ, while those with ASD have mean scores closer to 20 and above^63, 64^. Previous research suggests that mean scores on the SCQ are comparable in children with ASD across IQ strata, and it is valid for use in children with a mental age above 2.0^61^. For this analysis, we examined the total prorated scores on the 28 social interaction and communication items only, excluding the items pertaining to restricted/ repetitive behaviours. We did not adjust for IQ for this measure (see statistical analyses). The Reading the Mind in the Eyes Test (RMET) is a standardized test of social perception abilities^65^. Higher scores on the RMET are indicative of more accurate social perception. The child version of the RMET was used in this study, containing a total of 28 items^34^. Typically developing children tend to score between 15–20 out of 28 on this measure, while children with ASD tend to score 2–4 points lower than control subjects^25, 34^. RMET scores are known to vary with IQ^25^, therefore IQ was included as a covariate for these analyses. To more easily facilitate comparison of genotype effects across both measures, we report RMET scores with respect to the number of *incorrect* items. Therefore, throughout the manuscript, higher scores on either metric suggest greater social deficits. Other behavioural traits were quantified using the Child Behavior Checklist (CBCL) subscales^66–68^, and the Revised Children Anxiety and Depression Scale (RCADS)^69^.

### Selection of genetic markers

Four SNPs in *OXTR* from the third intronic region (rs53576, rs2254298, rs237887, rs13316193) were selected for genotyping based on existing literature at the time of study design suggesting an association between these markers and social abilities in participants with ASD or ADHD (Table S1). These SNPs, either alone or contributing to a haplotype, have also been associated with ASD risk^21, 31, 70–72^. Analysis for linkage disequilibrium (LD) was completed using Ldlink^33^, and the 1000 Genomes Project database of Caucasian samples (see Supplementary Table S3). Although rs13316193 and rs237887 showed moderate linkage, all four SNPs were sequenced to facilitate replication of previous findings within specific diagnostic groups. *AVPR1a* RS3 microsatellite length was sequenced as well, given initial findings suggesting an association between social abilities and genotype as well as receptor expression levels in typically developing subjects^36–38^, despite no association with social abilities in ASD in one study^29^.

### Genotyping

SNPs were analyzed on the MassARRAY Analyzer 4 system using iPLEX Gold chemistry (both Agena Biosciences, San Diego, CA, USA) using the primers in Supplementary Table S11 and the recommended manufacturer’s protocol. Genotypes were called using Typer 4.0 (Agena Biosciences). The *AVPR1A* RS3 polymorphism primers and methods are also listed in Table S11. Primers were adapted from that used by Tansey *et al*. to match the Human Feb. 2009 (GRCh37/hg19) assembly and to elevate the Tm but otherwise maintain the amplified position^73^. Results were analyzed using the software GeneMapper v. 3.7 (Life Technologies).

### Statistical analyses

All analyses were two-tailed, with the alpha set at 0.05, performed in SAS 9.3 (2002–2010, SAS Institute Cary, NC, USA). We used a logistic regression analysis to examine the proportion of items selected as ‘yes’ (for the 28 SCQ social communication items) or incorrect (for the 28 RMET items), using the PROC LOGISTIC function in SAS, which allows us to specify both the total score and the number of trials that this score is based on in the model outcome. This approach permitted adjustments for total number of items (e.g. decreasing the denominator for participants who answered >75% of items but did not complete the entire metric, n = 3 total). We adjusted for the contribution of the following predictor variables: genotype group, age, sex, ancestry (and IQ for analyses involving the RMET)^25^. Logistic regression was chosen for the main analyses given that scores on both metrics are sums of binary variables and therefore, follow a binomial distribution. Initial hypotheses were examined in the ASD group (for *OXTR* rs53576, rs2254298, and rs237887) and ADHD group (for rs53576, and rs1331619), including Caucasian participants only. We selected one specific categorical genotype risk pattern per SNP (i.e. dominant, recessive, or comparison of homozygous groups) for statistical testing based on the positive findings described in previous papers (Table S1). We subsequently combined data for both the ASD and ADHD groups (Caucasian only) and assessed the SNP*diagnosis interaction term to determine whether the magnitude of the genotype effect on social abilities varied by diagnosis. We present the odds ratios (ORs) of increasing SCQ/RMET scores for the hypothesized ‘high-risk’ vs. ‘low-risk’ genotype groups, based on the literature. That is, the odds of obtaining a positive/incorrect score on a single SCQ/RMET item for a participant in the ‘high-risk’ group divided by the odds of obtaining a positive/incorrect score for a participant in the ‘low-risk’ group. Thus, an odds ratio of 1 represents no risk group differences, while odds greater than 1 suggest increased risk. For presentation purposes, we also provide the predicted SCQ/ RMET scores by genotype group (by multiplying the predicted proportion of items selected or incorrect by the total number of items) adjusted to age 11.0 years and mean IQ scores. Details on covariates are provided in Supplementary Tables S8 and S9. Unadjusted p-values are reported; a Bonferroni correction for multiple comparisons is described alongside results. Finally, we combined data across ancestry groups and tested for genotype* ancestry interactions. Where non-significant (suggesting the effect of neuropeptide receptor genotype on social abilities does not vary by ethnicity), we pooled data across groups, added ancestry as a covariate (along with genotype, age, sex +/− IQ) and calculated the effect size (OR) in the larger varied ancestry group. For exploratory analyses involving anxiety, we compared genotype groups using ANOVAs; we adjusted for age and sex using ANCOVAs.

### Data availability

The datasets generated and analyzed during the current study are publicly available through Brain-CODE. http://www.braininstitute.ca/braincode/.

## Electronic supplementary material

Supplementary Information
