# Supplementary material for: Oxytocin Receptor Polymorphisms are Differentially Associated with Social Abilities across Neurodevelopmental Disorders
Source: Sci Rep. 2017 Sep 14;7:11618. doi: 10.1038/s41598-017-10821-0 (PMC5599599; doi:10.1038/s41598-017-10821-0)
Supplement: Supplementary file 1 — Supplementary Information [file 41598_2017_10821_MOESM1_ESM.pdf]

Supplementary Information

Oxytocin Receptor Polymorphisms are Differentially Associated with Social Abilities across Neurodevelopmental Disorders

Danielle A. Baribeau, M.D.,[1] Annie Dupuis, Ph.D.,[2] Tara A. Paton, Ph.D.,[3] Stephen W. Scherer, Ph.D.,[3,4] Russell J. Schachar, M.D.,[1,5] Paul D. Arnold, M.D., Ph.D.,[6] Peter Szatmari, M.D.,[1,5,7] Rob Nicolson, M.D.[8] Stelios Georgiades, Ph.D.,[9] Jennifer Crosbie, Ph.D.,[1,5] Jessica Brian, Ph.D.,[10,11] Alana Iaboni, Ph.D.,[11] Jason Lerch, Ph.D.,[12] Evdokia Anagnostou, M.D.[10,11*]

Author affiliations:

1. Department of Psychiatry, University of Toronto, Toronto, Ontario, Canada

2. Department of Biostatistics Design and Analysis, The Hospital for Sick Children, Dalla Lana School of Public Health, University of Toronto, Toronto, Ontario, Canada

3. The Centre for Applied Genomics, The Hospital for Sick Children, Toronto, Ontario, Canada

4. The McLaughlin Centre, University of Toronto, Toronto, Ontario, Canada

5. Department of Psychiatry, The Hospital for Sick Children, Toronto, Ontario, Canada

6. Hotchkiss Brain Institute, Departments of Psychiatry & Medical Genetics, University of Calgary, Calgary, Alberta, Canada

7. The Centre for Addiction and Mental Health, Toronto, Ontario, Canada

8. The Children's Health Research Institute and Western University, London, Ontario, Canada

9. Department of Psychiatry and Behavioural Neurosciences, McMaster University, Chedoke Hospital, Hamilton, Ontario, Canada

10. Department of Paediatrics, University of Toronto

11. Autism Research Centre, Bloorview Research Institute, Holland Bloorview Kids Rehabilitation Hospital, Toronto, Ontario, Canada

12. Program in Neuroscience and Mental Health, The Hospital for Sick Children, Department of Medical Biophysics, University of Toronto, Toronto, Canada

Corresponding Author: Dr. Anagnostou, Autism Research Centre, Bloorview Research Institute, Holland Bloorview Kids Rehabilitation Hospital, 150 Kilgour Road, Toronto, Ontario, Canada, M4G 1R8, Phone: 416-753-6005, Fax: 416-753-6046 eanagnostou@hollandbloorview.ca

Table S1: Literature review for studies examining phenotypic differences in social abilities as a continuous trait by neuropeptide receptor common variant, in populations with either ASD or ADHD

Study	N, Diagnosis	Common genetic variants tested	Continuous Metrics	Significant Findings	Survives correction for multiple comparisons?
**Yirmiya et al. 2006**[1]	128 with ASD, 217 parents, full phenotype data on 47 ASD participants only	*AVPR1a* microsatellites: RS1, AVR and RS3	ADOS, ADI-R, and VABS	Some individual associations with VABS. Some combined haplotypes associated with categorical grouping of scores on ADOS, ADI-R, VABS	NR
**Yrigollen et al. 2008**[2]	177 with ASD, 295 family members	3 *OXTR* SNPs (rs237885, rs2268493, rs237898)	ADOS, ADI-R, VABS	rs2268493 on some traits	No
**Lerer et al. 2008**[3]	152 with ASD, 266 parents, 146 siblings	18 *OXTR* SNPs (including rs13316193, and rs237887)	VABS	Nominal association of certain metrics with single SNPs, including rs2254298. Haplotype of rs237897-rs13316193-rs237889-rs2254298-rs2268494 associated with certain traits	Haplotype only
**Wermter et al. 2010**[4]	100 with ASD, 200 parents	22 *OXTR* SNPs (including rs13316193, rs53576, and rs2254298)	ADI-R	Some haplotypes (e.g. rs237851–rs6791619–rs53576–rs237884) associated with ADI-R subdomains	No
**Campbell et al. 2011**[5]	228-1738 with ASD who had continuous phenotype data	25 *OXTR* SNPs sequenced. Association with phenotype tested for rs2268493, rs1042778, and rs7632287 only	SRS, ADI-R and ADOS	Several associations between phenotype and rs2268493 and rs7632287 genotype detected	No
**Park et al. 2010**[6]	450 with ADHD and their parents, continuous phenotype data on 119 participants only	5 *OXTR* SNPs tested (rs6770632, rs237885, rs13316193, rs53576, rs237895)	SCDC	rs13316193 CC worse than TT (p=0.05), rs53576 AA better than AG (p=0.007)	Yes for rs53576 only
**Egawa et al. 2012**[7]	111 with ASD	14 *OXTR* SNPs tested,	CARS-TV	Rs237887 A-allele carriers showed lower imitation subscale scores	Yes
**Parker et al. 2014**[8]	79 with ASD, 52 siblings and 62 controls	*OXTR* rs53576 and rs2254298	SRS NEPSY-II, VABS-2	rs53576 AG/GG: worse affect recognition (p=.03)<br>rs2254298 AA/AG: worse on SRS (p=0.02)	NR but few tests
**Skuse et al. 2014**[9]	198 with ASD, 153 siblings, 311 parents	60 *OXTR* SNPs, and 31 *AVPR1a* SNPs	FMRT, GMT, FERT	rs237887 AA associated with worse face recognition memory (p=0.00015)	Yes
**Ayaz et al. 2015**[10]	99 ADHD, 99 controls	*OXTR* rs2268493, rs53576, rs13316193	SRS	None	No

Table S1 Legend: ADI-R: Autism Diagnostic Interview- Revised, ADOS: Autism Diagnostic Observation Schedule, VABS: Vineland Adaptive Behavior Scale, SRS: Social Responsiveness Scale, FRMT: Face Recognition Memory Test, GMT: Gaze Monitoring Test, FERT: Facial Emotion Recognition Test, NEPSY: A Developmental Neuropsychological Assessment, SCDC: Social and Communication Disorders Checklist, CARS-TV: Childhood Autism Rating Scale-Tokyo Version. ADHD: Attention Deficit Hyperactivity Disorder, ASD: Autism Spectrum Disorder. SNP: single nucleotide polymorphism, *AVPR1a*: arginine vasopressin receptor 1a, *OXTR*: oxytocin receptor. NR: not reported. Literature review examined studies published prior to September 2015 that tested for associations between neuropeptide receptor common genetic variants and phenotypic data as continuous traits in individuals with ASD or ADHD.

Table S2: ***OXTR*** **Genotype Frequencies**

	**Rs53576 (G/A) Minor: A**	**Rs237887 (G/A) Minor: G**	**Rs2254298 (G/A) Minor: A**	**Rs13316193 (G/A) Minor: C**
**Minor allele frequencies (N=617, 65% Caucasian)**				
**All Dx**	0.33	0.43	0.15	0.35
**ASD**	0.32	0.42	0.14	0.37
**ADHD**	0.34	0.45	0.17	0.31
**Reference genome minor allele frequencies (1000 genomes project, NIH.gov[11])**				
**All ethnicities**	0.39	0.40	0.21	0.40
**European ancestry**	0.35	0.46	0.11	0.38
**Non Caucasian**	0.40	0.38	0.23	0.41

Table S2 Legend: ASD: Autism spectrum disorder, ADHD: Attention deficit hyperactivity disorder

Table S3: Linkage Analysis

	rs2254298	rs13316193	rs53576
Rs237887	**D'= 0.917** R^2= 0.123	D'= **0.823** R^2= **0.352**	D'= 0.474 R^2= 0.142
Rs2254298		**D'= 1.0** R^2= 0.076	D'= 0.542 R^2= 0.02
Rs13316193			D'= 0.603 R^2= 0.119

Table S3 Legend: Ldmatrix for 5 *OXTR* SNPs from Caucasian participants in the 1000 genomes project.[11] SNP pairs where linkage detected in bold (D'> 0.8, R^2 >0.2).

Table S4: Hardy Weinberg Equilibrium Calculations

**SNP**	**Caucasian (n = 404)**		**Non-Caucasian (n = 213)**	
OXTR	**ASD (n=211)**	**ADHD (n=193)**	**ASD (n=130)**	**ADHD (n=83)**
**Rs53576**	0.06	0.7	0.2	0.1
**Rs2254298**	0.05	0.6	0.2	0.3
**Rs237887**	0.5	0.006	0.1	0.002
**Rs13316193**	0.2	0.4	0.4	0.2

Table S4 Legend: Hardy Weinberg Equilibrium p-values by diagnosis and ancestry, after comparing the observed and expected genotype frequencies using a chi-squared test (DF=1). ASD: Autism spectrum disorder, ADHD: Attention deficit hyperactivity disorder

Table S5: Correlations (r) between Measures in ASD

	IQ	CBCL-anxiety T-score	CBCL-anxiety total	SCQ total score	SCQ Soc Com items	RMET total score	RCADS total anxiety	RCADS social anxiety
**IQ**	1.00	0.10	0.14*	-0.21**	-0.31***	0.58***	0.22*	0.06
**CBCL-anxiety T-score**	0.10	1.00	0.94***	0.23***	0.12	0.06	0.55***	0.49***
**CBCL-anxiety total score**	0.14*	0.94***	1.00	0.24***	0.12*	0.03	0.63***	0.57***
**SCQ total score**	-0.21**	0.23***	0.24***	1.00	0.89***	-0.12	0.13*	0.26***
**SCQ Soc Com items**	-0.31***	0.12	0.12*	0.89***	1.00	-0.13	-0.01	0.15*
**RMET total score**	0.58***	0.06	0.03	-0.12	-0.13	1.00	0.18*	0.13
**RCADS total anxiety**	0.22*	0.55***	0.63***	0.13*	-0.01	0.18*	1.00	0.78***
**RCADS social anxiety**	0.06	0.50***	0.57***	0.26***	0.15*	0.13	0.78***	1.00

Table S5 Legend: IQ: Intelligence Quotient, CBCL: Child Behavior Checklist, SCQ: Social Communication Questionnaire, Soc Com: Social communication and interaction items only (repetitive behavior items excluded), RMET: Reading the Mind in the Eyes Test, RCADS: Revised Children's Anxiety and Depression Scale, ASD: Autism spectrum disorder, ADHD: Attention deficit hyperactivity disorder, *p<0.05; **p<0.001; ***p<0.0001

Table S6: Correlations (r) between Measures in ADHD

	IQ	CBCL-anxiety T-score	CBCL-anxiety total	SCQ total score	SCQ Soc Com items	RMET total score	RCADS total anxiety	RCADS social anxiety
**IQ**	1.00	-0.10	-0.10	-0.30**	-0.37***	0.25*	-0.17	-0.21
**CBCL-Anxiety T-score**	-0.10	1.00	0.98***	0.32***	0.19*	0.01	0.70***	0.60***
**CBCL-Anxiety total score**	-0.09	0.98***	1.00	0.32***	0.19*	0.04	0.69***	0.61***
**SCQ total score**	-0.30**	0.32***	0.32***	1.00	0.92***	-0.08	0.28**	0.28**
**SCQ SocCom items**	-0.37***	0.19*	0.19*	0.92***	1.00	-0.05	0.15	0.18*
**RMET total score**	0.25*	0.01	0.04	-0.08	-0.05	1.00	0.03	0.09
**RCADS total anxiety**	-0.17	0.67***	0.69***	0.30*	0.15	0.03	1.00	0.84***
**RCADS social anxiety**	-0.21	0.59***	0.61***	0.28*	0.18*	0.09	0.84***	1.00

Table S6 Legend: IQ: Intelligence Quotient, CBCL: Child Behavior Checklist, SCQ: Social Communication Questionnaire, Soc Com: Social communication and interaction items only (repetitive behavior items excluded), RMET: Reading the Mind in the Eyes Test, RCADS: Revised Children's Anxiety and Depression Scale, ASD: Autism spectrum disorder, ADHD: Attention deficit hyperactivity disorder, *p<0.05; **p<0.001; ***p<0.0001

Table S7: Mean SCQ and RMET incorrect scores by *OXTR* SNP genotype by Ancestry Group

OXTR SNPs and genotype		RMET Incorrect Responses						SCQ					
		**Caucasian**			**Non-Caucasian**			**Caucasian**			**Non-Caucasian**		
		n	LS Mean	(95% CI)	n	LS Mean	(95% CI)	n	LS Mean	(95% CI)	n	LS Mean	(95% CI)
**ASD**		**ASD**						**ASD**					
Rs53576	AA	17	10.4	(9.2-11.7)	5	10.8	(8.6-13.4)	27	11.5	(10.5-12.6)	12	13.8	(12.3-15.3)
	GA	50	11.1	(10.4-11.9)	31	13.3	(12.3-14.3)	78	13.3	(12.7-14.0)	63	14.8	(14.1-15.5)
	GG	64	11.4	(10.7-12.1)	23	11.4	(10.2-12.5)	102	14.2	(13.7-14.8)	50	16.1	(15.3-16.8)
Rs237887	AA	48	11.6	(10.8-12.4)	22	12.9	(11.7-14.0)	71	14.7	(14.0-15.3)	38	15.2	(14.3-16.0)
	GA	62	11.0	(10.4-11.7)	31	12.4	(11.3-13.4)	96	12.8	(12.2-13.3)	69	15.7	(15.0-16.4)
	GG	21	10.3	(9.1-11.5)	6	10.7	(8.7-12.8)	40	13.6	(12.7-14.5)	18	13.5	(12.3-14.8)
Rs2254298	AA	5	7.6	(5.8-9.8)	0			7	16.0	(13.9-17.9)	5	12.6	(10.3-15.0)
	GA	21	11.4	(10.2-12.6)	10	10.6	(9.0-12.3)	41	14.6	(13.7-15.4)	29	16.3	(15.3-17.3)
	GG	105	11.2	(10.6-11.8)	49	12.6	(11.8-13.4)	159	13.2	(12.8-13.7)	91	15.1	(14.5-15.7)
Rs13316193	CC	17	11.1	(9.8-12.4)	6	13.0	(10.8-15.2)	22	13.4	(12.3-14.6)	15	16.1	(14.7-17.4)
	CT	68	11.5	(10.8-12.2)	29	12.3	(11.2-13.4)	103	14.1	(13.6-14.7)	63	14.8	(14.1-15.5)
	TT	46	10.7	(9.9-11.5)	24	12.4	(11.3-13.5)	82	12.9	(12.3-13.5)	47	15.5	(14.7-16.3)
**ADHD**		**ADHD**						**ADHD**					
Rs53576	AA	6	13.4	(11.3-15.6)	3	13.1	(10.2-16.2)	21	5.7	(4.8-6.6)	10	4.3	(3.3-5.6)
	GA	34	11.0	(10.0-11.9)	21	11.0	(9.8-12.3)	81	5.0	(4.5-5.5)	46	5.6	(5.0-6.3)
	GG	33	10.9	(9.9-11.9)	12	11.4	(9.9-12.9)	90	4.0	(3.6-4.4)	26	4.9	(4.1-5.7)
Rs237887	AA	27	11.1	(10.0-12.1)	9	11.9	(10.1-13.8)	73	4.7	(4.3-5.2)	17	5.7	(4.8-6.8)
	GA	29	11.1	(10.1-12.2)	25	11.0	(10.0-12.1)	75	4.0	(3.6-4.5)	55	5.1	(4.5-5.7)
	GG	17	11.4	(10.1-12.7)	2	12.8	(9.1-16.6)	44	5.3	(4.7-6.0)	10	5.0	(3.9-6.4)
Rs2254298	AA	2	10.9	(7.5-14.7)	0			5	5.8	(4.2-7.8)	2	7.4	(4.7-11.1)
	GA	19	10.8	(9.6-12.1)	11	10.9	(9.3-12.6)	46	3.3	(2.8-3.8)	30	5.9	(5.2-6.8)
	GG	52	11.2	(10.4-12.0)	25	11.5	(10.3-12.6)	141	5.0	(4.6-7.3)	50	4.6	(4.1-5.2)
Rs13316193	CC	8	11.7	(9.8-13.7)	1	12.4	(7.7-17.6)	19	5.4	(4.5-6.4)	5	3.6	(2.4-5.3)
	CT	29	10.8	(9.8-11.8)	20	11.3	(10.1-12.5)	90	4.2	(3.8-4.6)	39	5.3	(4.6-6.0)
	TT	36	11.3	(10.3-12.3)	15	11.4	(10.0-12.8)	83	4.8	(4.4-5.3)	38	5.4	(4.7-6.1)

Table S7 Legend: LS mean: Least squares mean adjusted to age 11, and adjusted for sex, (and IQ on the RMET). 95% CI: confidence intervals. SCQ: Social Communication Questionnaire social communication/ interaction items (n= 28 items). RMET: Reading the Mind in the Eyes Test (n= 28 items). ASD: Autism spectrum disorder, ADHD: Attention deficit hyperactivity disorder.

Table S8: Details on Logistic Regression Model Covariates for Group of Participants with ASD

	SCQ				RMET Incorrect			
**SNP**	**Variable**	**Wald χ^2**	**OR (95% CI)**	**p-value**	**Variable**	**Wald χ^2**	**OR (95% CI)**	**p-value**
Rs53576	Sex (male vs. female)	0.05	1.0 (0.9-1.1)	0.8	Sex (male vs. female)	14.5	1.3 (1.1-1.5)	0.0001
	Age (per additional year)	99.9	1.06 (1.05-1.07)	<0.0001	Age (per additional year)	55.7	0.93 (0.92-0.95)	<0.0001
	Ancestry*SNP	0.7		0.4	Ancestry*SNP	0.5		0.5
	GG/GA (Other vs. Caucasian)		1.2 (1.1-1.4)	<0.0001	GG/GA (Other vs. Caucasian)		1.2 (1.1-1.4)	0.0009
	AA (Other vs. Caucasian)		1.4 (1.1-1.8)	0.01	AA (Other vs. Caucasian)		1.07 (0.7-1.6)	0.7
					IQ (per point)	181.2	0.98 (0.98-0.98)	<0.0001
Rs2254298	Sex (male vs. female)	0.04	1.0 (0.9-1.1)	0.8	Sex (male vs. female)	15.2	1.3 (1.2-1.5)	<0.0001
	Age (per additional year)	110.6	1.06 (1.05-1.07)	<0.0001	Age (per additional year)	60.1	0.93 (0.92-0.95)	<0.0001
	Ancestry*SNP	1.5		0.2	Ancestry*SNP	1.6		0.2
	GA/AA (Other vs. Caucasian)		1.2 (0.9-1.4)	0.1	GA/AA (Other vs. Caucasian)		1.0 (0.8-1.4)	0.8
	GG (Other vs. Caucasian)		1.3 (1.2-1.4)	<0.0001	GG (Other vs. Caucasian)		1.3 (1.1-1.5)	0.0003
					IQ (per point)	183.2	0.98 (0.98-0.98)	<0.0001
Rs237887	Sex (male vs. female)	0.4	1.05 (0.9-1.2)	0.5	Sex (male vs. female)	9.9	1.4 (1.1-1.7)	0.002
	Age (per additional year)	67.5	1.07 (1.05-1.09)	<0.0001	Age (per additional year)	28.4	0.93 (0.91-0.95)	<0.0001
	Ancestry*SNP	0.5		0.5	Ancestry*SNP	2.2		0.1
	AA (Other vs. Caucasian)		1.1 (0.9-1.3)	0.2	AA (Other vs. Caucasian)		1.3 (1.1-1.7)	0.01
	GG (Other vs. Caucasian)		1.01 (0.8-1.2)	0.9	GG (Other vs. Caucasian)		0.9 (0.6-1.5)	0.8
					IQ (per point)	90.4	0.98 (0.97-0.98)	<0.0001
Rs13316193	Sex (male vs. female)	1.5	1.1 (0.6-1.3)	0.2	Sex (male vs. female)	0.6	1.1 (0.9-1.3)	0.5
	Age (per additional year)	22.1	1.04 (1.02-1.05)	<0.0001	Age (per additional year)	33.7	0.92 (0.90-0.95)	<0.0001
	Ancestry*SNP	0.004		0.9	Ancestry*SNP	0.02		0.9
	CC (Other vs. Caucasian)		1.4 (1.1-1.8)	0.008	CC (Other vs. Caucasian)		1.4 (0.9-2.0)	0.1
	TT (Other vs. Caucasian)		1.4 (1.2-1.6)	<0.0001	TT (Other vs. Caucasian)		1.3 (1.1-1.7)	0.005
					IQ (per point)	50.5	0.98 (0.98-0.99)	<0.0001

Table S8 Legend: Logistic regression model covariates from aim 1; genotype effects are reported in main text. Outcome variables: SCQ: Social Communication Questionnaire, social communication/ interaction items only (n=28 items); RMET: Reading the Mind in the Eyes Test (n= 28 items); SNP: Single nucleotide polymorphism. Ancestry is classified as Caucasian (four Caucasian grandparents) or Non-Caucasian (Other) (see methods).

Table S9: Details on Logistic Regression Model Covariates for Group of Participants with ADHD

	SCQ				RMET Incorrect			
**SNP**	**Variable**	**Wald χ^2**	**OR (95% CI)**	**p-value**	**Variable**	**Wald χ^2**	**OR (95% CI)**	**p-value**
Rs53576	Sex (male vs. female)	24.7	1.5 (1.3-1.7)	<0.0001	Sex (male vs. female)	4.5	1.2 (1.0-1.5)	0.03
	Age (per additional year)	1.1	1.01 (0.99-1.03)	0.3	Age (per additional year)	76.8	0.84 (0.81-0.87)	<0.0001
	Ancestry*SNP	7.6		0.006	Ancestry*SNP	0.5		0.5
	GG/GA (Other vs. Caucasian)		1.2 (1.1-1.4)	0.001	GG/GA (Other vs. Caucasian)		1.0 (0.9-1.2)	0.8
	AA (Other vs. Caucasian)		0.7 (0.5-1.04)	0.07	AA (Other vs. Caucasian)		0.8 (0.5-1.4)	0.5
					IQ (per point)	31.4	0.98 (0.98-0.99)	<0.0001
Rs13316193	Sex (male vs. female)	15.7	1.5 (1.2-1.9)	<0.0001	Sex (male vs. female)	1.8	1.2 (0.9-1.6)	0.2
	Age (per additional year)	0.5	1.01 (0.98-1.04)	0.5	Age (per additional year)	48.3	0.83 (0.78-0.87)	<0.0001
	Ancestry*SNP	5.1		0.02	Ancestry*SNP	0.05		0.8
	CC (Other vs. Caucasian)		0.6 (0.4-1.0)	0.06	CC (Other vs. Caucasian)		*	*
	TT (Other vs. Caucasian)		1.1 (1.0-1.4)	0.2	TT (Other vs. Caucasian)		0.9 (0.8-1.3)	0.9
					IQ (per point)	15.0	0.99 (0.98-0.99)	0.0001
Rs2254298	Sex (male vs. female)	27.6	1.5 (1.3-1.7)	<0.0001	Sex (male vs. female)	5.2	1.2 (1.0-1.5)	0.02
	Age (per additional year)	1.01	1.01 (0.99-1.03)	0.3	Age (per additional year)	70.9	0.84 (0.81-0.87)	<0.0001
	Ancestry*SNP	29.1		<0.0001	Ancestry*SNP	0.3		0.6
	AA/GA (Other vs. Caucasian)		1.9 (1.5-2.4)	<0.0001	AA/GA (Other vs. Caucasian)		0.9 (0.7-1.3)	0.7
	GG (Other vs. Caucasian)		0.9 (0.8-1.1)	0.3	GG (Other vs. Caucasian)		1.04 (0.9-1.3)	0.7
					IQ (per point)	27.7	0.99 (0.98-0.99)	<0.0001
Rs237887	Sex (male vs. female)	12.9	1.5 (1.2-1.8)	0.0003	Sex (male vs. female)	0.2	1.1 (0.8-1.4)	0.7
	Age (per additional year)	1.5	0.98 (0.96-1.01)	0.2	Age (per additional year)	25.6	0.85 (0.80-0.91)	<0.0001
	Ancestry*SNP	2.2		0.1	Ancestry*SNP	0.09		0.8
	AA (Other vs. Caucasian)		1.2 (0.9-1.6)	0.1	AA (Other vs. Caucasian)		1.1 (0.8-1.6)	0.4
	GG (Other vs. Caucasian)		0.9 (0.7-1.3)	0.5	GG (Other vs. Caucasian)		1.04 (0.6-1.8)	0.9
					IQ (per point)	15.1	0.99 (0.98-0.99)	0.0001

Table S9 Legend: Logistic regression model covariates from aim 1; genotype effects are reported in main text. Outcome variables: SCQ: Social Communication Questionnaire, social communication/ interaction items only (n=28 items); RMET: Reading the Mind in the Eyes Test (n= 28 items); SNP: Single nucleotide polymorphism. Ancestry is classified as Caucasian (four Caucasian grandparents) or Non-Caucasian (Other) (see methods). *Odds ratios (OR) and confidence intervals (CIs) were not calculated, due to small sample size.

Table S10: Association between *OXTR* genotype and Social Deficits Using Additive Models in Caucasian Participants

Diagnosis	SNP (risk allele)	Measure	Wald χ^2	p-value	OR per copy of risk allele
ASD	rs53576 (G)	SCQ	22.4	<0.0001	1.2 (1.1-1.3)
		RMET	0.8	0.4	1.0 (0.9-1.1)
	Rs2254298 (A)	SCQ	12.1	0.0005	1.2 (1.1-1.3)
		RMET	7.7	0.006	0.8 (0.7-0.9)
	Rs237887 (A)	SCQ	7.8	0.005	1.1 (1.0-1.2)
		RMET	8.4	0.004	1.2 (1.0-1.3)
	Rs13316193 (C)	SCQ	5.0	0.03	1.1 (1.0-1.2)
		RMET	1.2	0.2	1.1 (1.0-1.2)
ADHD	rs53576 (G)	SCQ	17.9	<0.0001	0.8 (0.7-0.9)
		RMET	0.5	0.5	1.0 (0.8-1.1)
	rs2254298 (A)	SCQ	14.0	0.0002	0.8 (0.7-0.9)
		RMET	0.02	0.9	1.0 (0.8-2)
	rs237887 (A)	SCQ	1.1	0.3	1.0 (0.9-1.0)
		RMET	0.2	0.7	1.0 (0.9-1.1)
	rs13316193 (C)	SCQ	0.003	0.9	1.0 (0.9-1.1)
		RMET	0.02	0.9	1.0 (0.9-1.1)

Table S10 Legend: Models adjusted for age, sex, (and IQ on the RMET). 95% CI: confidence intervals. SCQ: Social Communication Questionnaire social communication/ interaction items. RMET: Reading the Mind in the Eyes Test, incorrect items. ASD: Autism spectrum disorder, ADHD: Attention deficit hyperactivity disorder.

Table S11: Primer Sequences

SNP	PCR primer 1	PCR primer 2	Extension primer
rs53576	ACGTTGGATGCTGTAGAATGAGCTTCCCAG	ACGTTGGATGGCACAGCATTCATGGAAAGG	CTGTGGGACTGAGGA
rs13316193	ACGTTGGATGTTGCTAATGGCACGTAGCAC	ACGTTGGATGATGTGTGTCAGGGAGGAGC	GGAGGACGGGAATGC
rs2254298	ACGTTGGATGCAGCTGAACATGGCAGCCT	ACGTTGGATGAGCATTCAGAGGAAGAAGCC	GAAACCATCCCTGTTTTC
rs237887	ACGTTGGATGACAACCTACCAGCCACAAAG	ACGTTGGATGTCTGTGAACTTCATCCCTGAT	AAGCTTTGCAATGAGGTAG

Table S11 Legend: Primer Sequences for OXTR Genotyping on MassARRAY Analyzer 4 System are listed in the table; underlined sequences are 5' non-template additions that are standard in the iPLEX protocol. PCR primers amplify the target variant and 80-100bp of flanking sequence; the extension primer flanks the variant and is extended by one nucleotide in the reaction. The *AVPR1A* RS3 polymorphism was amplified using primers 5'-CCTGTAGAGATGTAAGTGCTGCTCA-3' (forward) labeled with 6-FAM and 5'-TTTGGAAGAGACTTAGATGGTTCT-3' (reverse), which corresponds to hg19 genomic coordinates chr12:63,550,026-63,550,341. Primers were adapted from that used by Tansey et al. to match the Human Feb. 2009 (GRCh37/hg19) assembly and to elevate the Tm but otherwise maintain the amplified position.[12] 10 ul PCR reactions were performed containing 1ul of 2mM dNTPs, 1ul of 10X PCR buffer II (Life Technologies, Carlsbad, CA, USA), 0.49 ul of 25mM MgCl2, 0.1ul of AmpliTaq DNA polymerase (Life Technologies), 2ul of 5M betaine, 3.42 ul of water, 1ul of 10uM primer mix and 1ul genomic DNA (normalized to 50 ng/ul). The PCR cycling conditions were 10 min at 95°C followed by 30 cycles of 95°C (30 s), 60°C (30 s), 72°C (40 s) and a final extension step of 72°C for 10 min. Volumes of 1 μl of the PCR reactions were suspended in a 10 μl mixture of 20 μl GeneScan 500LIZ size standard in 980 μl Hi-Di formamide (Life Technologies). The samples were run on a 3730XL DNA Analyzer (Life Technologies) using the POP7 polymer and dye set G5. Results were analyzed using the software GeneMapper v. 3.7 (Life Technologies).

References

1 Yirmiya, N. *et al.* Association between the arginine vasopressin 1a receptor (AVPR1a) gene and autism in a family-based study: mediation by socialization skills. *Molecular psychiatry* **11**, 488-494, doi:4001812 [pii] 10.1038/sj.mp.4001812 (2006).

2 Yrigollen, C. M. *et al.* Genes controlling affiliative behavior as candidate genes for autism. *Biological psychiatry* **63**, 911-916, doi:10.1016/j.biopsych.2007.11.015 (2008).

3 Lerer, E. *et al.* Association between the oxytocin receptor (OXTR) gene and autism: relationship to Vineland Adaptive Behavior Scales and cognition. *Molecular psychiatry* **13**, 980-988, doi:4002087 [pii] 10.1038/sj.mp.4002087 (2008).

4 Wermter, A. K. *et al.* Evidence for the involvement of genetic variation in the oxytocin receptor gene (OXTR) in the etiology of autistic disorders on high-functioning level. *Am J Med Genet B Neuropsychiatr Genet* **153B**, 629-639, doi:10.1002/ajmg.b.31032 (2010).

5 Campbell, D. B. *et al.* Association of oxytocin receptor (OXTR) gene variants with multiple phenotype domains of autism spectrum disorder. *J Neurodev Disord* **3**, 101-112, doi:10.1007/s11689-010-9071-2 (2011).

6 Park, J. *et al.* Evidence that genetic variation in the oxytocin receptor (OXTR) gene influences social cognition in ADHD. *Prog Neuropsychopharmacol Biol Psychiatry* **34**, 697-702, doi:10.1016/j.pnpbp.2010.03.029S0278-5846(10)00122-3 [pii] (2010).

7 Egawa, J. *et al.* Association between OXTR and clinical phenotypes of autism spectrum disorders. *Psychiatry research* **208**, 99-100, doi:10.1016/j.psychres.2012.11.007 (2013).

8 Parker, K. J. *et al.* Plasma oxytocin concentrations and OXTR polymorphisms predict social impairments in children with and without autism spectrum disorder. *Proceedings of the National Academy of Sciences of the United States of America* **111**, 12258-12263, doi:10.1073/pnas.1402236111 1402236111 [pii] (2014).

9 Skuse, D. H. *et al.* Common polymorphism in the oxytocin receptor gene (OXTR) is associated with human social recognition skills. *Proceedings of the National Academy of Sciences of the United States of America* **111**, 1987-1992, doi:10.1073/pnas.1302985111 1302985111 [pii] (2014).

10 Ayaz, A. B. *et al.* Oxytocin system social function impacts in children with attention-deficit/hyperactivity disorder. *Am J Med Genet B Neuropsychiatr Genet* **168**, 609-616, doi:10.1002/ajmg.b.32343 (2015).

11 Machiela, M. J. & Chanock, S. J. LDlink: a web-based application for exploring population-specific haplotype structure and linking correlated alleles of possible functional variants. *Bioinformatics* **31**, 3555-3557, doi:10.1093/bioinformatics/btv402 (2015).

12 Tansey, K. E. *et al.* Functionality of promoter microsatellites of arginine vasopressin receptor 1A (AVPR1A): implications for autism. *Molecular autism* **2**, 3, doi:10.1186/2040-2392-2-3 2040-2392-2-3 [pii] (2011).
